# *Photobacterium damselae* subsp. *damselae* in Stranded Cetaceans: A 6-Year Monitoring of the Ligurian Sea in Italy

**DOI:** 10.3390/ani14192825

**Published:** 2024-09-30

**Authors:** Roberta Battistini, Chiara Masotti, Federica Giorda, Carla Grattarola, Simone Peletto, Camilla Testori, Simona Zoppi, Enrica Berio, Maria Ines Crescio, Nicola Pussini, Laura Serracca, Cristina Casalone

**Affiliations:** 1 Istituto Zooprofilattico Sperimentale del Piemonte, Liguria e Valle d’Aosta, Via Bologna 148, 10154 Torino, Italy; roberta.battistini@izsto.it (R.B.); chiara.masotti@izsto.it (C.M.); federica.giorda@izsto.it (F.G.); carla.grattarola@izsto.it (C.G.); simone.peletto@izsto.it (S.P.); camilla.testori@izsto.it (C.T.); simona.zoppi@izsto.it (S.Z.); mariaines.crescio@izsto.it (M.I.C.); nicola.pussini@izsto.it (N.P.); cristina.casalone@izsto.it (C.C.); 2 ASL 1 Sistema Sanitario Regione Liguria, Via Aurelia Ponente 97, 18038 Sanremo, Italy; e.berio2@asl1.liguria.it

**Keywords:** dolphin, *Stenella coeruleoalba*, *Tursiops truncatus*, polymerase chain reaction, *Photobacterium damselae*, hemolysin

## Abstract

**Simple Summary:**

*Photobacterium damselae* subsp. *damselae* (Pdd) is a marine bacterium that can infect a variety of marine animals and humans. Although this bacterium has been isolated from several stranded dolphins and whales, its pathogenic role in cetaceans is still unclear and very limited information exists on its occurrence in these animals. In this study, we report data relating to the presence of Pdd in marine mammals stranded within the Liguria Pelagos Sanctuary from 2017 to 2022. Our findings show a 41.5% (22/53) Pdd prevalence in stranded cetaceans, where 22.7% (5/22) of these were positive in at least one of the Pdd virulence factors which, in previous studies, have been related to Pdd pathogenicity. Our results also revealed that in all cases where cetaceans tested positive for Pdd, other well-known pathogens for these species were also present. This finding supports the hypothesis that Pdd is an opportunistic agent that might contribute to the worsening of health conditions in subjects already compromised by other pathogens, contributing to their death. However, further studies are necessary to investigate and deepen this hypothesis.

**Abstract:**

*Photobacterium damselae* subsp. *damselae* (Pdd) is an increasingly common bacterium in post-mortem diagnostics of beached marine mammals, but little is known about its precise etiological responsibility. To estimate the prevalence of Pdd in stranded cetaceans from 2017 to 2022 on the Ligurian coast (Pelagos Sanctuary), we tested tissues from 53 stranded individuals belonging to four cetacean species. DNA extracts from cetacean tissue were screened using a polymerase chain reaction (PCR) assay targeting the Pdd *ureC* gene. Positive samples were screened by PCR for *dly, hlyA_pl_* and *hlyA_ch_* hemolysin genes, which were confirmed by sequencing. Twenty-two out of 53 (41.5%) cetaceans analyzed by PCR were confirmed for Pdd DNA in at least one tissue among those analyzed. Five of these cetaceans were positive for at least one of the hemolysin genes tested. In all Pdd-positive cetaceans, other pathogens that were considered responsible for the *causa mortis* of the animals were also found. The results provide new information on the spread of Pdd in cetaceans and support the thesis that Pdd might be an opportunistic agent that could contribute to worsening health conditions in subjects already compromised by other pathogens. However, further studies are needed to investigate and deepen this hypothesis.

## 1. Introduction

*Photobacterium damselae* subsp. *damselae* (Pdd) is a halophilic gram-negative bacterium within the family *Vibrionaceae*. Like other *Vibrionaceae* species, its natural habitat is the aquatic ecosystem [1,2]. Pdd is considered a primary pathogen for a wide range of aquatic species, including molluscs, crustaceans, reptiles, and, more frequently, aquaculture fish [1,3,4,5]. Its role as a minor zoonotic agent, responsible for severe necrotizing fasciitis in humans, is also known [6,7,8]. The pathogenicity of Pdd has been correlated to its ability to produce several virulence proteins, such as hemolysins and histamine, and to the presence of an iron uptake system [9,10]. In particular, it has been reported that hemolytic strains produce a chromosome-encoded hemolysin, named HlyA_ch_, and highly hemolytic strains also harbor the virulence plasmid pPHDD1, which encodes for two other different hemolysins—damselysin (Dly) and HlyA_pl_ [10]—and was recently renamed phobalysin (PhlyP) [11]. The entry route of this bacterium in animal species is still poorly investigated. However, there is evidence that the infection is transmitted through water in the presence of skin lesions as a port of entry [1,12]. Pdd can survive in seawater and sediments for a long period, maintaining infectivity and pathogenic properties [13]. New host individuals can be infected through water when temperature and salinity are favorable [12,14,15]. The spread of the disease appears to depend mainly on water temperature; in fact, some reports have associated the presence of Pdd epidemics in fish with unusual increases in water temperature [16,17,18]. Therefore, the occurrence of diseases caused by this pathogen is likely to increase in the future due to the expected increase in seawater temperature resulting from global climate change [19]. 

In cetaceans, this bacterium has been isolated from healthy and stranded dolphins and whales [20,21], and it is commonly considered an opportunistic agent without a clear explanation of its possible pathogenic role [22]. In recent years, its presence has been more frequently identified in stranded cetaceans of the Mediterranean Sea [22,23,24,25,26]. The increase in infection reports may be due to increased surveillance efforts focused on stranding cetaceans, improved diagnostic approaches, or increased disease prevalence. Understanding the impacts of disease in cetacean populations relies on postmortem diagnosis of disease limited by the small number of carcasses recovered. The occurrence of Pdd in marine mammals remains a poorly understood phenomenon, since very little information exists. In this work, data relating to the presence of Pdd in marine mammals stranded in the Liguria Pelagos Sanctuary from 2017 to 2022 are reported. 

## 2. Materials and Methods

### 2.1. Samples Collection

Between January 2017 and December 2022, 53 cetaceans were stranded and found dead along the Ligurian coast of Italy. The cetaceans included 37 striped dolphins (*Stenella coeruleoalba*), 13 bottlenose dolphins (*Tursiops truncatus*), two Cuvier’s beaked whales (*Ziphius cavirostris*), and one pilot whale (*Globicephala melas*). The animals were examined and submitted to complete postmortem examination, routine pathological analysis, and cause-of-death assessment by the Italian National Reference Center for diagnostic activities in stranded marine mammals (C.Re.Di.Ma.) according to standard protocols [27]. Investigations also regarded the presence of relevant pathogens, including bacterial, viral, fungal, protozoan, and helminthic agents. Bacteriological analyses were performed on all main organs onto a non-selective blood-based agar medium (BAB, Liofilchem Italy s.r.l.), and confirmation of grown bacterial species was initially performed by using matrix-assisted laser desorption ionization time-of-flight mass spectrometry (MALDI-TOF MS) according to the manufacturer’s protocol. Specific bacteriological procedures were also applied in order to screen for *Listeria* spp., *Salmonella* spp., and *Brucella* spp. [28]. Carcass recovery information and individual data of each stranded cetacean were also recorded. All these data are available in the annual stranding reports of C.Re.Di.Ma. [29,30,31,32,33,34]. A few cases have also been previously published [35,36,37,38,39,40]. 

In particular, for Pdd detection, when available, spleen, liver, lymph nodes, lung, kidney, and brain samples were collected during the routine postmortem investigations and submitted for biomolecular analysis in parallel with the bacteriological analysis already described above. The presence of virulence factors was also investigated by biomolecular methods in the cetaceans that tested positive for Pdd in cetaceans. The presence of macro and micro hemorrhagic lesions was also assessed. The procedure for the biomolecular identification of Pdd and virulence factors is described below.

### 2.2. DNA Extraction 

A small portion of each tissue sample (25–30 mg) was added to sterile phosphate buffered saline (pH 7.2) (300 µL) and then physically disrupted on a TissueLyser II homogenizer (Qiagen, Hilden, Germany) by high-speed stirrer in plastic tubes with stainless steel beads (5 mm in diameter). An aliquot of the resulting supernatant (200 μL) from each sample was used to extract DNA using the IndiSpin Pathogen Kit (Indical Bioscience, Leipzig, Germany), as described in the manufacturer’s instructions, and the eluted nucleic acids (100 μL) were stored at −20 °C until use. 

### 2.3. PCR Amplification of Pdd 

Genome amplification for the detection of Pdd was performed using a PCR that amplifies a 448 bp fragment of *ureC* gene [41]. The PCR assays used a reaction solution containing PCR buffer 1× (Invitrogen, Waltham, MA, USA), 2.5 mmol·L–1 MgCl_2_ (Invitrogen, Waltham, MA, USA), 0.2 mmol·L–1 dNTPs (Sigma-Aldrich, St Louis, MO, USA, 1 µmol·L–1 of primers (Metabion International Ag, Planegg, Germany), 1 U Platinum Taq DNA polymerase (Invitrogen, Waltham, MA, USA), 2 μL of DNA template, and ultra-pure water up to 50 μL. Amplification conditions were as follows: 2 min at 94 °C, 50 cycles of 30 s at 95 °C, 30 s at the primer annealing temperature (65 °C), 1 min at 72 °C, and a final extension of 10 min at 72 °C. Reactions were carried out in a C1000 Touch thermal cycler (Bio-Rad, Hercules, CA, USA). The negative and positive controls (DNA of Pdd ATCC 33539) were included in each amplification series. The PCR products were analyzed by electrophoresis on 2% agarose gel (Sigma-Aldrich, St Louis, MO, USA) containing Gel Green Nucleic Acid Gel Stain 10,000× (Biotium, Fremont, CA, USA) in comparison with molecular weight markers, and then photographed on a Gel-Doc UV transilluminator system (Bio-Rad, Hercules, CA, USA). 

### 2.4. PCR Amplification of Hemolysin Genes 

Samples testing positive for Pdd were screened by PCR for hemolysin genes (*dly*, *hlyA_pl_,* and *hlyA_ch_*) using primers and thermal cycling conditions as previously described [42]. The amplification reactions for *dly* and *hlyA_ch_* genes were performed in a final volume of 25 µL containing 0.625 U of Platinum Taq DNA polymerase (Invitrogen, Waltham, MA, USA), 400 nM of each primer, 1× PCR Buffer, 3 mM MgCl_2_, 0.4 mM dNTPs, 1 µL of DNA sample, and sterile water to volume. The PCR for *hlyA_pl_* gene was performed using a mixture containing 1×PCR buffer, 3 mM MgCl_2_, 0.2 mM dNTPs each, 1 µM of each primer, 1 U of Platinum Taq DNA polymerase (Invitrogen, Waltham, MA, USA), 2 µL of the DNA sample, and water to a final volume of 50 µL. All reactions were carried out in a C1000 Touch thermal cycler (Bio-Rad, Hercules, CA, USA) and PCR products were separated by electrophoresis on 2% agarose gel stained with 10,000× Gel Green Nucleic Acid Gel Stain (Biotium, Fremont, CA, USA) and visualized under ultraviolet (UV) illumination. The presence of hemolysin DNA in a sample was indicated by the presence of a band of the appropriate size 549 bp (*dly*), 767 bp (*hlyA_pl_*), and 353 bp (*hlyA_ch_*) with concordant negative and positive controls. 

### 2.5. Sequencing

Amplification products were purified with the QIAquick Gel Extraction kit (Qiagen) and sequenced using a BrilliantDye Terminator v.3.1 kit (NimaGen, Nijmegen, The Netherlands) according to the manufacturer’s instructions. The amplicons were purified with an AutoSeq G-50 Dye Terminator Removal kit (GE Healthcare, Chicago, IL, USA) and run on a SeqStudio Genetic Analyzer (Applied Biosystems, Foster City, CA, USA). Obtained forward and reverse sequences were aligned using the SeqMan software from the Lasergene package v. 7.2 (DNASTAR Inc., Madison, WI, USA). The resulting consensus sequences were aligned to detect any nucleotide variation and matched with available sequences retrieved from the National Center for Biotechnology Information (NCBI) database using the BLAST tool against the sequence records available in GenBank to confirm species identification.

### 2.6. Data Analysis

Data on the date of stranding, species, and positivity found in different organs were subjected to descriptive analysis. Subsequently, a univariate analysis was performed using the chi-square test to assess whether there were differences in prevalence among species and among tissues in the individuals which tested as Pdd positive. Finally, the presence of infection seasonality was assessed by analysis-of-variance (ANOVA).

## 3. Results

Between January 2017 and December 2022, samples from 53 cetaceans stranded on the Ligurian coastline (Italy) were tested for Pdd DNA by PCR. A total of 272 tissue samples were analyzed. Twenty-two animals out of 53 (41.5%) tested positive for Pdd DNA in at least one tissue, including the brain, lung, lymph nodes, kidney, liver, and spleen (Table 1 and Table 2). All positive samples were confirmed by sequencing. The sequence analysis revealed that almost all *ureC* and all hemolysin gene sequences shared 100% nucleotide identity when compared to each other as well as the sequence records available in GenBank. The only exception was the *ureC* sequence of sample ID 6, which showed 92.4% similarity with the other sample sequences. Moreover, microbiological examinations allowed the isolation of Pdd from seven out of 19 (36.8%) of the tested animals, mainly from the lung, lymph nodes, and brain samples (Table 3). The chi-square test showed no differences when Pdd prevalences were compared between tissues in positive animals. 

Of the 22 positive cetaceans, 15 were striped dolphins, and seven were bottlenose dolphins (Table 1). The chi-square test showed no differences when Pdd prevalences were compared between striped dolphins and bottlenose dolphins. 

Three PCR-positive cetaceans were stranded, respectively, in 2017, 2018, 2020, 2021, and 2022, and seven were stranded in 2019 (Table 1). Most positive cetaceans were stranded during summer, followed by winter, spring, and autumn (Table 3), but no seasonality was found in the Pdd prevalence according to statistical analysis.

Of the five cetaceans PCR-positive for the *hlyA_ch_* hemolysin gene, one was also positive for *hlyA_pl_* and *dly* hemolysin genes. The *hlyA_pl_* and *dly* genes were detected only in one out of 22 (4.5%) cetaceans testing positive to Pdd (Table 1). Only one cetacean (4.5%) was positive for all three of the considered genes in the lymph node, while the spleen was positive for *hlyA_ch_* and *dly* together (Table 2). Hemorrhagic lesions were observed in tissue samples, which resulted in a positive for hemolysins only in samples ID 16 and ID 17 (Table 3). 

Of the 22 PCR-positive cetaceans, 11 were adults, five were sub-adults, four were juveniles, and two were calves (Table 3). Sex was not determined in one animal, while five were female and 16 were male. Among the 22 cetaceans that tested positive for Pdd, other pathogen microorganisms were found in co-presence. Cetacean Morbillivirus (CeMV) was the most prevalent (10/22), followed by herpesvirus (7/22), *Toxoplasma gondii* (5/22), and in fewer numbers of *Salmonella* 1,4,[5],12:i:-(2/22), *Clostridium perfringens* (2/22), and others (refer to Table 3). Therefore, in 13 of these animals (59%), the *causa mortis* after necroscopy was correlated to an infectious origin. In some of the six animal tissues (ID: 5, 6, 11, 16, 17, 20, 22) that tested positive for the molecular investigation of Pdd, hemorrhagic lesions were also present mainly in the lymph nodes. In two of these cases (ID 16 and 22), where the Pdd was isolated, the pathological condition found was compatible with the presence of Pdd hemolytic strain and *Clostridium sordelli* in one case, and Pdd and *Brucella* spp. in the other case (Table 3). 

## 4. Discussion

Our study presents available data on Pdd prevalence in cetaceans stranded during the last six years along the Ligurian coastline, part of the Pelagos Sanctuary. 

The prevalence of Pdd over such a long period of time has never been reported in marine mammals except for one study [21] that found 4% of cetaceans positive for Pdd from the northeast USA and 8% from cetaceans stranded along southwest Florida Gulf coasts [21]. In fact, positivity for Pdd is usually reported in cetaceans linked to a single stranding [23,24] or, in some cases, associated with mass mortality events [22,43]. Casalone et al. 2014 [22] reported a Pdd prevalence of 62% of the stranded dolphins in an unusual mortality event that occurred along the Tyrrhenian Sea coast of Italy during the first three months of 2013. 

In our study, Pdd DNA was detected by PCR in 41.5% of cetaceans stranded from January 2017 to December 2022, and 22.7% (5/22) of these PCR-positive cetaceans were also positive for at least one of the hemolysin genes tested. In particular, four out of five (80%) cetaceans showed positivity only for the *hlyA_ch_* gene, which seems to be the one with the lower pathogenicity based on experimental studies conducted on mice [10]. In one out of five (20%) cetaceans positive for Pdd virulence factors, the co-presence of the three hemolysins investigated was found, a condition correlated to the highest level of mortality in experimental animals. Previous investigations have demonstrated that strains of Pdd characterized by greater virulence correspond to those capable of inducing more significant hemorrhagic lesions in fish, thus establishing a relationship between hemolysis and virulence [44]. On the contrary, in the matrices of our study in which high and medium pathogenic hemolysins (dly and hlyA_pl_) were confirmed, no hemorrhagic lesions were observed either at a macroscopic or histopathological level. However, in a previous report, strains of Pdd having the chromosomal *PhlyC* gene (*hlyA_ch_*), in the absence of the *dly* gene, caused severe chronic suppurative pneumonia in dead dolphins [23], supporting previous indications that this virulence factor is not essential for pathogenesis [45]. Furthermore, it should be considered that Pdd is a pathogen mainly for fish, while the potential to cause disease in humans and mammals is an accidental condition. A recent study shows that mammalian body temperature represents a stress condition for this microorganism, unlike that of fish, which are cold-blooded animals. When Pdd grows at 37 °C, the genes involved in virulence are repressed, including iron acquisition systems, and the virulence factor *dly* is four-fold downregulated at this temperature [46].

Analysis of *ureC* gene sequences revealed that most positive samples shared the same nucleotide sequence. However, this sequence showed 100% identity with only three records in GenBank, specifically three Pdd isolates from South Korea (Acc. Nos. CP035458, CP021152, CP063050) collected in Yangtze finless porpoises (*Neophocena asiaorientalis*) and Beluga whale (*Delphinapterus leucas*) in 2017 and 2020, respectively. The *ureC* of sample ID 6, however, was different from the other sequences (92.4% similarity), but a number of 100% identical records were found in the GenBank database. Most of the Pdd strains identified by molecular testing were not identified by culture tests. In healthy carriers, the bacterial load can persist in low numbers, and it is therefore very difficult to isolate them with classical culture methods [47]. Furthermore, the isolation of Pdd when it grows at mammalian body temperature can be difficult as already reported by another study [46]. In fact, the stress conditions at this temperature compromise the cells’ vitality and shape control [46]. PCR has been able to detect the presence of DNA of these microorganisms even when present in low numbers and significantly reduce identification times. However, it does not provide us with information on the microorganisms’ viability and is not able to test phenotypic hemolytic activity. The rapidity of PCR testing is a potential advantage over cultures. Molecular methods maintain an advantage, at least theoretically, over time, and they are more sensitive than cultures. Furthermore, this technique can produce a more accurate and feasible diagnosis [48,49,50,51]. Therefore, by combining the two techniques, it is possible to obtain as much information as possible about the bacterial strain. 

The present study also examined several different tissue samples for each cetacean. This extensive analysis shows that most of the animals were infected in multiple tissues at the same time, with the lymph node being the most frequently infected tissue

In our study, the hypothesis of *causa mortis* in 13 of the 22 cetaceans positive for Pdd was correlated to an infectious origin, mainly of a viral and bacterial nature, and, to a lesser extent, a parasitic one. In two cases, an anthropic cause related to fishery interaction was identified; however, it was in the presence of underlying pathologies. It is interesting to note that, in all cases where cetaceans tested positive for Pdd, other well-known pathogens for these species, such as CeMV, *T. gondii*, and α-herpesvirus, were also present. These pathogens are responsible for serious systemic infections in the examined subjects, often accompanied by severe histopathological lesions.

The coinfection of Pdd and other pathogens has been previously described in stranded cetaceans [22,24,52], and CeMV-induced immunosuppression has been suggested to act as a predisposing factor to Pdd infections [43].

The prevalence of Pdd in live cetaceans has never been directly investigated. However studies on the microbial community profile in dolphins reveal the presence of this bacterium also in healthy cetaceans [53,54]. In a study of wild dolphins from the Gulf of Mexico and two Atlantic Ocean locations, *V. damselae* (later renamed Pdd) resulted in the most commonly recovered bacteria (64.1% of all animals tested) from both anal/fecal and blowhole samples [54]. 

Numerous other aerobic microorganisms of clinical significance were also isolated from healthy cetacean samples [21,53,54,55]. Other studies indicate that debilitated animals are characterized by a greater number of opportunists than healthy animals [21]. Therefore, based on existing studies, many potential pathogens occur in a commensal or transient state in healthy wild dolphin populations. To better investigate this aspect, it would therefore be appropriate for future research to perform molecular or cultural screening for Pdd not only in deep invasive tissue samples, but also in non-invasive samples such as skin, blowhole, genitalia, rectum, or feces of stranded cetaceans. The common presence of coinfection in stranded dolphins, as highlighted in our study, would seem to support the hypothesis that in the case of a weakening of the immune system of cetaceans due to other infections or other causes, these opportunistic pathogens may take over, increasing the state of debilitation of the individual with consequent death and/or stranding. 

Dolphins are gregarious animals and move over large distances, which makes it possible to disseminate bacteria to other individuals of the same social group and perhaps to other groups, in new geographic areas and, perhaps, to new animals. The dissemination of these pathogens in the marine environment also represents a potential threat to public health since Pdd causes infections also in humans [1], and Pdd has been associated with drug resistance in aquaculture [56] and in human clinical settings [57]. This fact highlights the importance of monitoring the presence of this and other pathogens in marine mammals and deepening the analyses with further information, such as the acquisition of the presence of antibiotic resistance genes in Pdd strains isolated from cetaceans, since, at the moment, there are no data in the literature.

## 5. Conclusions

The pathogenic role of Pdd has not been clarified yet in cetaceans [22,24]; however, the presence of virulence factors, and not just the identification of the bacterium, in association with important cetacean pathogens could lay the ground for evaluating the role of Pdd in the worsening of conditions already characterized by serious impairment. However, further studies are necessary to investigate and deepen this initial hypothesis. 

## Figures and Tables

**Table 1:** Pdd and hemolysins DNA presence in cetaceans stranded on the Ligurian coast (from 2017 to 2022).

Year	Total Animals Tested	Pdd		Species (No. Pdd Positive; %)	Pdd Positive/Haemolysin Positive (Type)
		No.	%		
2017	5	3	60.0	*Stenella coeruleoalba* (3/5; 60.0)	3/0
2018	9	3	33.3	*Stenella coeruleoalba* (3/8; 27.5)	3/0
				*Tursiops truncatus* (0/1; 0.0)	-
2019	11	7	63.6	*Stenella coeruleoalba* (4/6; 66.7)	4/0
				*Tursiops truncatus* (3/4; 75.0)	3/0
				*Globicephala melas* (0/1; 0.0)	-
2020	11	3	27.3	*Stenella coeruleoalba* (2/9; 22.2)	2/1 (*hlyAch*)
				*Tursiops truncatus* (1/1; 100.0)	1/1 (*hlyAch*; *hlyApl*; *dly*)
				*Ziphius cavirostris* (0/1; 0.0)	-
2021	11	3	27.3	*Stenella coeruleoalba* (1/5; 20.0)	1/1 (*hlyAch*)
				*Tursiops truncatus* (2/6; 33.3)	2/1 (*hlyAch*)
2022	6	3	50.0	*Stenella coeruleoalba* (2/4; 50.0)	2/0
				*Tursiops truncatus* (1/1; 100.0)	1/1 (*hlyAch*)
				*Ziphius cavirostris* (0/1; 0.0)	-
Total	53	22	41.5	*Stenella coeruleoalba* (15/37; 40.5)	22/5 (5 *hlyA_ch_*; *1 hlyA_pl_*; *1 dly*)
				*Tursiops truncatus* (7/13; 53.8)	
				*Ziphius cavirostris* (0/2; 0.0)	
				*Globicephala melas* (0/1; 0.0)	

**Table 2:** Pdd and hemolysins DNA presence in tissues of cetaceans stranded on the Ligurian coastline (Italy) from 2017 to 2022.

Tissue Type	Total Tested	Pdd		Haemolysin Positive/Pdd Positive	
		No.	%	No. and Type	%
Spleen	41	9	22.0	2/9 (1 *hlyAch*; *dly*)	22.2
				(1 *hlyAch*)	
Liver	46	11	24.0	0/11	0.0
Lymph node(s)	47	16	34.0	3/16 (1 *hlyAch*; 1 *hlyApl*; 1 *dly*)	18.7
				(2 *hlyAch*)	
Lung	50	14	28.0	3/14 (*hlyA_ch_*)	21.4
Kidney	45	7	15.6	1/7 (*hlyA_ch_*)	14.3
Brain	42	9	21.4	2/10 (*hlyA_ch_*)	20.0
Total	272	66	24.3	11/67 (11 *hlyA_ch_*; 1 *hlyA_pl_*; 2 *dly*)	14.9

**Table 3:** Pathogen microorganisms found in stranded cetaceans recovered from the Ligurian coastline from 2017 to 2022 that tested positive for Pdd and hemolysin DNA, Pdd culture, and cause of death after necroscopy. M: male, F: female, n.d: not determined, J: juvenile, A: adult, S.A.: sub-adult, C: newborn-calf. n.t.: not tested.

ID	Laboratory Reference	Stranding Date	Species (Sex and Age Class)	Tissue Pdd PCR + (Hemolysin)/ Tissue Culture +	Tissue Positive for Pdd with Hemorrhagic Lesions	Other Microorganisms	Cause of Death	Ref.
1	16769	16 February 2017	*S. coeruleoalba* (M, J)	Liver, lung, brain/-	-	CeMV, *Brucella ceti,* *T. gondii*, *Sarcocystis* spp., *Monorygma* sp. (cysts)	Bacterial/viral/ parasitic infection	[29,35,36,37]
2	78983	14 September 2017	*S. coeruleoalba* (F, A)	Kidney/-	-	CeMV, α-herpesvirus, *Salmonella* 1,4,[5],12:i:-, *T. gondii*, *Phyllobotrium* sp., *Monorygma* sp., *Pennella* sp.	Bacterial/viral/ parasitic infection	[29,36,37,38]
3	90704	16 October 2017	*S. coeruleoalba* (F, A)	Liver, lymph nodes, lung, brain/-	-	α-herpesvirus	Viral infection	[29]
4	5129	20 January 2018	*S. coeruleoalba* (M, A)	Liver, kidney, brain/-	-	CeMV, α-herpesvirus, *Salmonella* 1,4,[5],12:i:-, *Phyllobotrium* sp., *Monorygma* sp.	Bacterial/viral infection	[30,38,39]
5	5386	21 January 2018	*S. coeruleoalba* (M, A)	Liver, lung/-	Liver	CeMV, ƴ-herpesvirus, *Salmonella tsevie*, pulmonar nematodes (*Pseudaliidae*), *Pholeter* sp. (cysts), *Phyllobotrium* sp., *Monorygma* sp.	Viral infection	[30]
6	87558	29 October 2018	*S. coeruleoalba* (F, J)	Spleen, liver, lymph nodes, lung, kidney/-	Lung	CeMV, *Listeria innocua*	Bacterial/viral infection	[30,37,39]
7	18013	23 February 2019	*T. truncatus* (M, J)	Lymph nodes, lung/-	-	α-herpesvirus, *Enterococcus faecium*, *Staphylococcus* spp., mycetes (mucorales)	n.d.	[31,40]
8	21724	5 March 2019	*S. coeruleoalba* (F, A)	Lymph nodes, lung/lung	-	CeMV, *Moraxella* spp., *Phyllobotrium* sp. (cysts), *Monorygma* sp. (cysts)	Viral infection	[31,37,39]
9	33212	4 April 2019	*S. coeruleoalba* (M, J)	Lung, kidney/n.t.	-	Pulmonar nematodes, *Pholeter* sp. (cysts)	n.d	[31]
10	42472	7 May 2019	*T. truncatus* (M, A)	Brain/n.t.	-	CeMV	Anthropic cause: vessel collision	[31,39,40]
11	59260	4 July 2019	*T. truncatus* (M, A)	Spleen, liver, lymph nodes, lung, brain/-	Brain	CeMV, *T. gondii*	Bacterial/viral/ parasitic infection	[31,37,39,40]
12	62877	21 July 2019	*S. coeruleoalba* (M, A)	Spleen, lymph nodes, lung, kidney, brain/-	-	CeMV, *Phyllobotrium* spp. (cysts), *Monorygma* spp. (cysts)	Bacterial/viral/ parasitic infection	[31,39]
13	63558	23 July 2019	*S. coeruleoalba* (M, C)	Spleen, liver, lymph nodes, lung, brain/-	-	*T. gondii*	Bacterial/parasitic infection	[31]
14	51352	13 July 2020	*T. truncatus* (M, A)	Spleen (hlyAch; dly), lymph nodes (hlyAch; hlyApl; dly), lung (hlyAch), brain(hlyAch)/liver, lymph nodes, lung, brain	-	*T. gondii*, α-herpesvirus, *Listeria grayi*, *C. perfringens*, *Penicillium* spp.	Anthropic cause: by catch (consequence of underlying pathologies)	[32,36,40]
15	55654	30 July 2020	*S. coeruleoalba* (M, C)	Spleen, liver, lymph nodes/brain	-	-	Natural cause	[32]
16	60669	23 August 2020	*S. coeruleoalba* (M, S.A)	Spleen (hlyAch), liver, lymph nodes (hlyAch), kidney (hlyAch)/lymph nodes, brain	Lymph nodes	*Clostridium sordelli*, *Phyllobotrium* sp.	Bacterial infection	[32]
17	6128	22 January 2021	*S. coeruleoalba* (M, A)	Lymph nodes, lung (hlyAch)/lymph nodes, lung	Lung	α-herpesvirus, *Pholeter* sp.(cysts), *Campula* spp. *Phyllobotrium* sp. (cysts), *Monorygma* sp.	Natural cause	[33]
18	30434	29 March 2021	*T. truncatus* (M, A)	Lymph nodes (hlyAch)/lymph nodes, lung, brain	-	*Listeria seeligeri*, *Carnobacterium* spp., Cetacean poxvirus 1, *Phyllobotrium* sp., Intestinal trematodes, *Serratia* spp., *Pholeter* sp.	Anthropic cause: by catch (consequence of underlying pathologies)	[33]
19	73951	10 September 2021	*T. truncatus* (F, S.A)	Lymph nodes, lung/-	-	*T. gondii*, CeMV, *Erysipelothrix rhusiopathiae, Phyllobotrium* sp., *Pholeter* sp.	n.d.	[33,39,40]
20	8319	30 January 2022	*S. coeruleoalba* (M, S.A)	Spleen, liver, lymph nodes/-	Lymph nodes	α-herpesvirus, *C. perfringens, Monorygma* sp., *Pholeter* sp.	Bacterial/viral infection	[34]
21	63831	2 August 2022	*T. truncatus* (M, S.A)	Lymph nodes, lung (hlyAch), brain (hlyAch)/n.t.	-	*Pholeter* sp.	n.d.	[34]
22	85889	24 October 2022	*S. coeruleoalba* (n.d., S.A)	Spleen, liver, lymph nodes, kidney, brain/kidney	Lymph nodes, brain	*Brucella* spp., *Pholeter* sp., *Phyllobotrium* sp.	Bacterial infection	[34]

## Data Availability

All data supporting the present study are reported in this study. Further inquiries can be directed to the corresponding author.
